# A global survey of adverse event following immunization surveillance systems for pregnant women and their infants

**DOI:** 10.1080/21645515.2016.1175697

**Published:** 2016-05-09

**Authors:** Christine Cassidy, Noni E. MacDonald, Audrey Steenbeek, Justin R. Ortiz, Patrick L. F. Zuber, Karina A. Top

**Affiliations:** a School of Nursing, Faculty of Health Professions, Dalhousie University, Halifax, Nova Scotia, Canada; b Department of Pediatrics, Dalhousie University, Halifax, Nova Scotia, Canada; c Canadian Center for Vaccinology, IWK Health Center, Halifax, Nova Scotia, Canada; d Initiative for Vaccine Research, World Health Organization, Geneva, Switzerland

**Keywords:** adverse event following immunization, neonate, pregnancy, surveillance, vaccination

## Abstract

Strengthening antenatal care as a platform for maternal immunization is a priority of the World Health Organization (WHO). Systematic surveillance for adverse events following immunization (AEFI) in pregnancy is needed to identify vaccine safety events. We sought to identify active and passive AEFI surveillance systems for pregnant women and infants. Representatives from all National Pharmacovigilance Centers and a convenience sample of vaccine safety experts were invited to complete a 14-item online survey in English, French or Spanish. The survey captured maternal immunization policies, and active and passive AEFI surveillance systems for pregnant women and infants in respondents' countries. The analysis was descriptive. We received responses from 51/185 (28%) invited persons from 47/148 (32%) countries representing all WHO regions, and low, middle and high-income countries. Thirty countries had national immunization policies targeting pregnant women. Eleven countries had active surveillance systems to detect serious AEFI in pregnant women and/or their infants, including six low and middle-income countries (LMIC). Thirty-nine countries had passive surveillance systems, including 23 LMIC. These active and passive surveillance programs cover approximately 8% and 56% of the worldwide annual birth cohort, respectively. Data from one active and four passive systems have been published. We identified 50 active and passive AEFI surveillance systems for pregnant women and infants, but few have published their findings. AEFI surveillance appears to be feasible in low and high resource settings. Further expansion of AEFI surveillance for pregnant women and sharing of vaccine safety information will provide additional evidence in support of maternal immunization policies.

## Introduction

Vaccinations during pregnancy can protect the mother and newborn from the effects of severe infection.^1^ Several immunizations are currently recommended in pregnancy, including seasonal influenza, tetanus toxoid (TT) and tetanus-diphtheria-acellular pertussis vaccine (Tdap).^2-5^ Additionally, some vaccinations have been used in pregnant women in certain circumstances where the benefits may outweigh any theoretical risks, such as meningococcal conjugate serogroup A vaccine (MenAfriVac).^2^ However, maternal immunization programs have not been established in many countries, and where they do exist, vaccine uptake may be low.^6-8^

The World Health Organization (WHO) is supporting efforts to advance the maternal immunization agenda.^9^ A potential barrier to immunization is vaccine hesitancy among patients and health care providers, which in some cases, is related to concerns about vaccine safety. To date, most published studies on vaccine safety in pregnant women have been from high-income countries.^10^ There are limited pre- or post-licensure data on the safety of vaccines used primarily or exclusively in pregnant women in low and middle-income countries. Additionally, there are unique safety concerns in this population due to potential risks to the developing fetus, not just the pregnant woman. These factors highlight the importance of robust adverse event following immunization (AEFI) surveillance systems to detect rare and serious adverse events in pregnant women and their offspring.^1,11^

In previous work, we conducted a systematic review of the English–language literature to identify published reports of active and passive AEFI surveillance systems for pregnant women and their offspring.^10^ Sixteen articles met the criteria for review, and one additional article was identified after publication.^10,12^ Twelve articles were from the United States and the remaining five were from Belgium, France, Australia, Sweden, and Taiwan, respectively. The articles described a variety of observational and descriptive studies that used active and passive surveillance methods to collect data on the safety of several vaccines including: pandemic and seasonal influenza vaccines, hepatitis A, human papillomavirus, meningococcal conjugate, smallpox, Tdap, and varicella vaccines. The review did not identify any published reports of ongoing AEFI surveillance systems outside of the United States that specifically focus on pregnant women or their offspring.^10,12^

We hypothesized that there might be additional AEFI surveillance systems that capture events in these populations that have not been reported in the literature. We therefore sought to identify active and passive AEFI surveillance systems for pregnant women and their offspring in WHO member countries. The results of this study could then be used to develop recommendations for improving AEFI surveillance and collection of safety data for maternal immunization programs.

## Results

Of the 154 National Pharmacovigilance Center (NPC) representatives invited to participate in the survey (representing 144 countries with NPCs that had email contact information), we received responses from 41 participants representing 39 countries. Following a second round of survey invitations to an additional 31 individuals (4 of whom were from countries not included in the first round), we received responses from 7 participants representing an additional 6 countries for a total of 47 countries. Participants from Argentina, China, and Sweden submitted their responses via email as they were unable to respond online.

In total, 51 participants in 47 countries completed the survey for an overall response rate of 32% [47/148 of countries surveyed; 51/185 (28%) total invitees]. Two responses were received from each of four countries (Australia, Norway, Sudan, and United Kingdom). Twenty-five respondents (49%) were national regulators, followed by public health officials (9/51, 18%), and immunization program managers (6/51, 12%). The remaining 11 respondents (22%) included other representatives from NPCs and academic researchers. Seven individuals opened the survey and consented to take part but did not submit any responses to the questions; they were not considered respondents and were excluded from the analysis.

The countries represented by the partial or complete responses received are shown in Table 1. We received responses from low, middle and high-income countries and from all WHO regions. Response rates were 40% (19/48) among high-income countries, 31% (23/74) among middle-income countries, and 21% (5/24) among low-income countries (p = 0.3). Response rates among WHO regions ranged from 39% in the European region to 24% in the African region, but the differences were not statistically significant (p = 0.8). Responses were received from 9 of the 20 most populous countries in the world (China, India, United States, Brazil, Pakistan, Mexico, Philippines, Germany, and Democratic Republic of Congo).

**Table 1.** Summary of survey responses by country.

Country	National Maternal Immunization Policy Exists	Active AEFI surveillance: Maternal	Active AEFI surveillance: Infant	Passive AEFI surveillance	Passive: ascertains pregnancy status	Registry for unintentional immunization
High-Income^13^						
Australia	X	X		X		
Canada				X		
Chile	X			X		
Czech Republic				X		
Denmark	X			X		
Finland	X			X	X	
Germany	X			X	X	
Ireland	X			^*^		
Israel	X			X		
Latvia				X	X	
Netherlands			X	X		
Norway	X			X	X	
Oman	X	X				
Singapore				X		
Slovenia				X		
Sweden	X			X		
Switzerland	X			X		
United Kingdom	X	X	X	X	X	X
United States	X	X	X	X		X
Middle Income^13^						
Anguilla	X			X		
Argentina	X			X	X	X
Brazil	X			X	X	X
Bulgaria						
Cameroon				X		
China	X			X	X	
Costa Rica	X	X	X	X		
Côte d'Ivoire	X					
Ghana				X		
Hungary			X	X	X	
India	X			X		
Jordan	X					
Kyrgyzstan				X		
Mexico	X	X	X	X	X	
Montenegro				X		
Morocco						
Pakistan	X			X		
Philippines	X			X	X	
Saint Vincent and the Grenadines	X			X	X	
Senegal	X			X		
Serbia				X	X	
Sudan	X	X	X	X	X	
Thailand				X		
Low-income^13^						
Burkina Faso		X		X		
Cambodia	X	X		X	X	
Congo, the Democratic Republic of the	X			X	X	
Gambia	X					
Mozambique				X	X	

*Note.* AEFI, adverse event following immunization

* No national AEFI surveillance system per WHO criteria, but some AEFI are captured in the national monitoring system for serious adverse drug reactions.

### Maternal immunization policies

Respondents from 30/47 countries noted that their country has a national policy recommending routine immunization of pregnant women with one or more vaccines (Table 1). Respondents from eight countries reported that there was no policy, and nine respondents did not know if there was a policy. Among the countries with a policy, the following immunizations were recommended in pregnancy under certain circumstances: influenza vaccination (14 countries), tetanus toxoid (14 countries), tetanus-diphtheria-acellular pertussis (9 countries), hepatitis B (2 countries) and meningococcal conjugate vaccine (1 country). Of the 30 countries with a national immunization policy targeting pregnant women, 28% (8/30) have an active AEFI surveillance program that captures serious AEFI in pregnant women or their infants and 80% (24/30) have a passive AEFI surveillance program. In addition, 3/8 (38%) countries with no immunization policies regarding pregnant women had active surveillance systems and 5/8 (62%) had passive systems.

### Active surveillance

In total, 11 active surveillance systems to detect serious AEFI in pregnant women or their infants were identified across all country income levels and 5 of 6 WHO regions (Tables 1 and 2). Five countries had national active AEFI surveillance systems to detect AEFI in both pregnant women and infants, and two countries had national active AEFI surveillance systems to detect outcomes in pregnant women only. Four countries had active AEFI surveillance systems in one or more districts of the country to detect serious AEFI in pregnant women and/or infants. Additionally, the respondent from Singapore reported that active AEFI surveillance is conducted on an *ad hoc* basis in that country [e.g., during 2009 influenza A (H1N1) pandemic]. For those countries identified as having specific active national and/or regional AEFI surveillance systems for pregnant women and their infants, the program name and publication details (if provided by respondents) are shown in Table 3.

**Table 2.** Active and passive adverse event following immunization surveillance systems by country income level and WHO region.^13,14^

		Active surveillance			
	Countries from which responses were received	Maternal	Infant	Passive surveillance	No system/Unknown
	n	n	n	n	n
Income Level					
High	19	4	3	16	2
Middle	23	3	4	19	4
Low	5	2	0	4	1
WHO Region					
The Americas	9	3	3	9	0
European	18	1	3	15	3
African	8	1	0	6	2
South-East Asian	2	0	0	2	0
Eastern Mediterranean	5	2	1	2	2
Western Pacific	5	2	0	5	0

**Table 3.** Additional information provided on national and/or regional active adverse event following immunization surveillance systems for pregnant women and/or their infants.

Country	WHO Region^14^	Income Level^13^	Program Name	Published Results
Netherlands	European	High	pREGnant	No
United States	Americas	High	Vaccine Safety Datalink	Yes^15^
Australia	Western Pacific	High	FASTMum	No
Costa Rica	Americas	Middle	Programa ampliado de Inmunizaciones y Centro Nacional de Farmaco-vigilancia	No
Mexico	Americas	Middle	Red Negativa	No
Cambodia	Western Pacific	Low	National Immunization Program	No

Respondents from six countries reported that plans are underway to implement active surveillance programs to capture outcomes in pregnant women and/or their offspring: Argentina (in 2015/2016), Democratic Republic of the Congo (in 2016), Netherlands (in 2016), Ghana, Mozambique, and Pakistan (dates not specified). An active surveillance system is being established in seven centers in Argentina as part of a Pan American Health Organization project. In the Netherlands, a pregnancy drug registry will be implemented in 2016 that will capture adverse outcomes in infants exposed to medications and vaccines *in utero*.

### Passive surveillance

In most countries (39/47) there is a national passive AEFI surveillance program that encompasses any vaccine and any vaccine recipient (Tables 1 and 2). Respondents from three countries reported that they did not know if there is a national passive AEFI surveillance program in their country, and one additional respondent noted that AEFI were occasionally reported to a national adverse drug reaction database but there was no dedicated AEFI surveillance program. In 17 of the 39 countries with passive systems (44%), the AEFI reporting form asks if the event occurred in a pregnant woman. In eight countries, specific analyses of existing passive surveillance data have been conducted or are planned to assess the risk of AEFI in pregnant women and/or their infants with the following target vaccines: influenza, Tdap, tetanus toxoid, and hepatitis B. Four countries have published the results of their analyses.^16-19^

### Unintentional immunization in pregnancy

In four countries (Argentina, Brazil, United Kingdom, United States) there are specific vaccine registries or passive surveillance systems that capture outcomes of women and their offspring when a vaccine was administered without knowledge of ongoing pregnancy (Table 1).

### Population coverage

Passive AEFI surveillance systems were found in 39 countries whose birth cohorts comprise 56% (73,602,000/131,000,000) of the worldwide annual birth cohort (See Supplemental Figure 1).^20,21^ National or regional active AEFI surveillance systems for maternal and/or infant outcomes were present in 11 countries whose birth cohorts comprise 8% (10,483,000/131,000,000) of the worldwide birth cohort.

### Qualitative analysis

Respondents from 17 countries provided additional comments regarding AEFI surveillance for maternal immunization programs. Most provided specific information regarding activities in their countries (included above where appropriate). Respondents from six countries (all middle-income countries) provided more general comments about AEFI surveillance. Two themes emerged from their comments: need for improved surveillance, and need for support with research and capacity building. In regards to the first theme, one respondent stated: “AEFI surveillance of pregnant women and the infant outcomes needs improvement and better analysis” while another stated that “there is the dire need that active as well as passive surveillance must be conducted in pregnant mothers.” Two respondents expressed interest in participating in international initiatives to improve AEFI surveillance.

## Discussion

This global survey of active and passive AEFI surveillance systems for pregnant women and their infants identified 10 active and 35 passive surveillance systems that have not been previously reported in the literature. The majority of countries represented in this survey have national policies for routine immunization of pregnant women, underscoring the need for robust AEFI surveillance systems that specifically target pregnant women and their infants. AEFI surveillance systems capturing outcomes in pregnant women were reported by respondents in low, middle and high-income countries across all WHO regions, suggesting that AEFI surveillance is feasible in both high and lower resource settings. However, due to the low response rate (32% of invited countries), we were unable to fully ascertain the prevalence of maternal immunization programs globally or to evaluate potential barriers to AEFI surveillance among pregnant women and their offspring. Nonetheless, the findings highlight ongoing vaccine safety surveillance activities for pregnant women and their infants in a broad cross-section of countries, and identify gaps in surveillance and reporting that can be the target of future interventions. The paucity of publications from the surveillance programs identified in this survey underscore the need to develop a mechanism and process to facilitate harmonized reporting and pooling of data on vaccine safety in pregnant women and their offspring. The low survey response rate may suggest a general lack of AEFI surveillance capacity globally, particularly in low and middle-income countries (LMIC), as previously reported.^22,23^ In countries with limited capacity to identify AEFI in their childhood immunization programs, surveillance for AEFI in their maternal immunization programs is likely to be even more limited. Expansion of AEFI surveillance capacity in maternal immunization programs requires support with infrastructure and training, as emphasized by several of the respondents in this study and reported by others.^22^

Active AEFI surveillance programs to detect serious adverse events in mothers and/or infants were reported by participants in 11 countries. However, together these countries represent only 8% of the worldwide birth cohort and some programs were limited to specific vaccines (e.g., influenza) or specific regions of a country. Therefore, the ability of these systems to reliably detect very rare (<1/100,000) or previously unreported adverse events occurring in vaccinated women is limited, highlighting the need to strengthen and expand active surveillance programs. Such efforts should focus initially on specific countries with the interest and capacity, as well as sufficient numbers of births, to reliably and precisely detect pre-specified increases in rates of target AEFI over the background rate.

Capacity to improve active AEFI surveillance exists in a number of middle and high income countries that have introduced nation-wide integrated health information systems (IHIS) in recent years. In Belize, the IHIS includes disease prevention and management algorithms for eight health domains, including maternal health.^24^ By capturing every encounter with the public healthcare system, including outpatient and non-physician visits (e.g., public health nurse), outcomes of vaccinated pregnant women and their infants can be evaluated with minimal additional infrastructure. Sharing of analytic protocols between countries with similar systems could further increase capacity for safety evaluation. St Vincent and the Grenadines, one of the countries reporting in this survey, also has a similar IHIS to Belize.(Michael Graven, personal communication)

National passive AEFI surveillance systems were in place in 39/47 countries that contributed to the survey. However, only 44% (17/39) of passive systems specifically captured pregnancy status on the reporting forms, and data have been published from only four countries (China, Norway, Sweden, US).^16-19^ Furthermore, three of the reports cited by survey participants related to pandemic influenza vaccines which are not currently in use.^16-18^

To increase the value of established passive AEFI surveillance systems for maternal immunization programs, a query about pregnancy status needs to be added to all existing reporting forms. This is especially important for countries with large birth cohorts that are statistically better placed to detect very rare adverse events. For countries without passive AEFI surveillance programs (including 7/47 countries in this survey), action is needed. Passive surveillance systems are necessary in all countries to support their immunization programs, including their maternal immunization programs.

Argentina, Brazil, UK, and US reported having vaccine registries or surveillance systems that capture outcomes of women who unintentionally received a vaccine during pregnancy, and their infants. Many of these programs have focused on specific vaccines such as rubella (during a mass immunization campaign in Brazil),^25^ measles-mumps-rubella, varicella, and human papillomavirus (Vaccine in Pregnancy program in the UK),^26^ and smallpox (US).^10^ There appear to be gaps in surveillance of some vaccines that are contraindicated in pregnancy (e.g., yellow fever, rabies vaccines). Existing registries that capture inadvertent immunizations in pregnancy should be expanded to capture all vaccines that are currently contraindicated in pregnancy. Additionally, such registries should be established in more countries so more safety and outcome data can be collected.

Based on these findings, we recommend several measures for improving AEFI surveillance in maternal immunization programs. First, capacity for active surveillance can be built into existing health systems, particularly in countries with integrated electronic health information systems. Second, to improve capture of AEFI in pregnant women, countries with passive surveillance systems should consider adding a question about pregnancy status to their routine AEFI surveillance reporting forms. Third, all countries should be supported in the establishment of a process for causality assessment of serious AEFI in pregnancy and reporting of findings. Although not assessed in this study, causality assessment is an important aspect of AEFI surveillance. Fourth, a mechanism and process are needed to facilitate harmonized reporting and pooling of data on vaccine safety in pregnant women and their offspring on a global level. Finally, surveys of active and passive surveillance for AEFI in pregnant women and their infants should be repeated regularly to verify if improvement has occurred.

Our survey had limitations. Only WHO member states that had a NPC with email contact information were represented and the 31 immunization experts who were invited to participate represented a convenience sample. There was also likely a degree of response bias. There were only five respondents from low-income countries and as a result, the findings are not likely to be representative of all low-income countries in all regions. Countries that lack AEFI surveillance systems were probably less likely to respond and we cannot draw conclusions about the barriers to expanding surveillance in those countries. Additionally, we may not have identified all existing AEFI surveillance systems targeting pregnant women. Because it was a self-administered survey, there may have been reporting errors among respondents and respondents may not have had complete knowledge of the surveillance activities in their country. Although the survey was available in three languages (French, English, and Spanish), respondents with an alternative first language may not have understood all questions correctly. Finally, there were seven individuals who consented to participate and opened the survey but did not answer any of the questions. It is unknown if this was due to technical or language issues with the survey or Internet connection, the respondents had nothing to report, and/or no knowledge of the surveillance activities in their country.

### Conclusions

This survey identified a range of ongoing AEFI surveillance activities across all 6 WHO regions. However, few have published findings. It is imperative that countries with existing maternal/infant vaccine safety data be encouraged to analyze their data and publish their results. The qualitative comments suggest help with analysis may be necessary for some. Further expansion of active and passive AEFI surveillance for pregnant women and infants, together with regular surveys to verify progress, are also needed. Collection and reporting of safety data is an important strategy to support trust and confidence in immunization of pregnant women.

## Methods

### Study design and subjects

We contacted all National Pharmacovigilance Centers to participate in the survey. NPCs are WHO-approved pharmacovigilance centers participating in the WHO Program for International Drug Monitoring and are usually a part of, or closely linked to, the national drug regulatory agency.^27,28^ The survey was self-administered online and was available in English, French and Spanish. Invitations with an embedded personalized link to the survey were sent via email. Reminder emails were sent three weeks and ten weeks after the initial invitation. Following the second reminder email, separate invitations were sent to a convenience sample of 31 individuals who were known to members of the research team as key personnel knowledgeable about AEFI surveillance in their respective countries.

### Ethics

This study received ethical approval from the Dalhousie University Research Ethics Board and WHO Research Ethics Review Committee (#RPC684).

### Survey instrument and development

The investigators developed the survey and individuals with expertise in immunization program management and vaccine safety reviewed it for content validity. The survey comprised 14 questions; both multiple-choice and open-ended free text questions (see Supplemental Content 1). Questions included the respondent's country and role in immunization, current national maternal immunization programs, and recent, current, and planned active and passive AEFI surveillance systems. Definitions of active and passive surveillance were provided. The survey was developed in English, professionally translated into French and Spanish, and back translated. Participants indicated their consent to the survey on the introductory page in order to proceed to the survey.

Opinio survey software version 6.9.1 (ObjectPlanet, Oslo, Norway) was used on a server hosted in Halifax, NS, Canada. The Opinio survey software maintains rigorous security and prevents participants from taking the survey more than once.

### Statistical analysis

Datasets were downloaded from Opinio into SPSS version 21 (IBM, Armonk, NY) for analysis. Participants who indicated their consent to participate and submitted a response to at least one survey question were counted as “respondents” in the analysis. The data were analyzed by: a) presence of a national immunization policy that included pregnant women; b) country income level based on the World Bank ranking;^13^ c) WHO Region;^14^ and d) coverage of the worldwide birth cohort.^20,21^ The analysis was descriptive. Chi-squared tests were used in analysis of proportions. The free text responses were analyzed qualitatively by 2 co-authors (KAT and CC) who identified patterns in responses and grouped similar responses together.

If two or more responses were received from the same country (i.e. more than one respondent invited), they were reviewed in tandem for congruence. If conflicting information was provided, differences were reconciled bearing in mind that the objective was to detect as many surveillance programs as possible involved in the review and approval of the manuscript.

## Supplementary Material

Supplementary files
