# Supplementary material for: A global survey of adverse event following immunization surveillance systems for pregnant women and their infants
Source: Hum Vaccin Immunother. 2016 May 9;12(8):2010–6. doi: 10.1080/21645515.2016.1175697 (PMC4994761; doi:10.1080/21645515.2016.1175697)
Supplement: Supplementary files [file khvi-12-08-1175697-s001.zip › 2016HV0028R-s02.docx]

**WHO Survey on Adverse Events Following Immunization (AEFI) Surveillance for Pregnant Women**

**1.** Country your institution serves: ______________________________

Add drop down menu

2. Correspondent’s role in immunization

a) immunization program manager

b) national regulator

c) public health official

d) other, specify general category__________________

**4.** Does the country your institution serves have a national policy recommending routine immunization of pregnant women with one or more vaccines?

Yes No Don’t Know

**If yes, for which vaccine(s)? Please tick If Recommended are they supplied free? Please Tick**

|  | Recommended | Supplied Free |
| --- | --- | --- |
| Influenza |  |  |
| Tetanus toxoid |  |  |
| Tdap or pertussis vaccine |  |  |
| MenA conjugate |  |  |
| Other – please specify |  |  |

**Active Adverse Events Following Immunization (AEFI) Surveillance:**

**Active surveillance: means that**

1) there is regular contact with health care providers who actively search for cases of adverse events following immunization and

2) that there is analysis and comparison of the incidence of specific events in vaccinated and unvaccinated populations to allow for early detection of a problem

**5. Using the above definition,** is there a national system in the entire country your institution serves dedicated /specific to **active AEFI surveillance** to detect serious AEFI in pregnancy and/ or in the infant(s) of the pregnancies where the woman received a vaccine?

For maternal AEFIs /outcomes Yes No Don’t know

if yes, when did it begin, year?

infant outcomes of the pregnancy Yes No Don’t Know

if yes, when did it begin, year?

**6. Using the above definition,** is there an **active AEFI surveillance** network to detect serious AEFI in pregnancy and/ or in the infant(s) of the pregnancy where woman received a vaccine only in a region(s) or in specific area(s) in the country your institution serves?

For maternal AEFI/outcomes Yes No Don’t know

if yes, when did it begin, year?

infant outcomes of the pregnancy Yes No Don’t know

if yes, when did it begin, year?

**7.**  If you have a specific active **national AEFI surveillance** for pregnant women and their infants - in the country your institution serves please give name of the program _______________________________________________

Have the results of this program ever been published in any format? Yes No

If yes, are they available on line or in a medical journal?

If yes, where can they be found? ____________________________

and any other comments______________________________________________

8. If **no** national maternal specific active AEFI program exists in your country, is one being planned?

to detect maternal AEFI/outcomes Yes No Don’t know

if yes, when is the program expected to begin? ______ (dropdown menu for year)

to detect infant outcomes of the pregnancy Yes No Don’t know

if yes, when is the program expected to begin? ______ (dropdown menu for year)

**Passive AEFI Surveillance:**

**Passive surveillance**: means there is spontaneous adverse event following immunization reporting by health care providers or individuals

9. Is there a national **passive AEFI general surveillance program** for all vaccines and all vaccine recipients- in the country your institution serves (i.e. not just for pregnant women)?

Yes No Don’t know

**10.** If yes, does the country’s **passive AEFI surveillance program** specifically ask **if the event occurred in a pregnant woman?**

Yes No Don’t know

if yes, when did this begin, year?

If yes and reporting form is online - please give url ___________________

11**. If you answered yes to question 10,** has the country your institution serves done or are planning to do specific analyses to assess the risk of AEFI in pregnant women with one or more vaccines with **data from the passive AEFI system** (e.g. an analysis of safety of pandemic 2009 influenza vaccine etc)?

maternal AEFI/outcomes Yes No Don’t know

infant outcomes of the pregnancy Yes No Don’t know

If yes, also please note vaccine(s): _____________________________________

If yes, have the data been published or reported? _______________If so, where? __________________________

12. Does the country your institution serves have a **vaccine registry** that captures outcomes of mothers (and their infants) who ***unintentionally*** received a vaccine during pregnancy **i.e.** (**i.e. the health care worker who gave the vaccine did not know that the woman was pregnant)?**

maternal AEFI/outcomes Yes No Don’t know

infant outcomes of the pregnancy Yes No Don’t know

**13.** If yes, where are these registry data regularly published or reported?

_________________________________

**Both Active and Passive AEFI Surveillance of pregnant women**

**14.** Is there anything else about AEFI surveillance of pregnant women and the infant outcomes of the pregnancy in the country your institution serves that you would like to add? ___________________________________________

Thank you

All those on the distribution list for this survey will be emailed a summary of the findings.
