# Supplementary material for: A global survey of adverse event following immunization surveillance systems for pregnant women and their infants
Source: Hum Vaccin Immunother. 2016 May 9;12(8):2010–6. doi: 10.1080/21645515.2016.1175697 (PMC4994761; doi:10.1080/21645515.2016.1175697)
Supplement: Supplementary files [file khvi-12-08-1175697-s001.zip › 2016HV0028R-s03.pptx]

## Slide 1
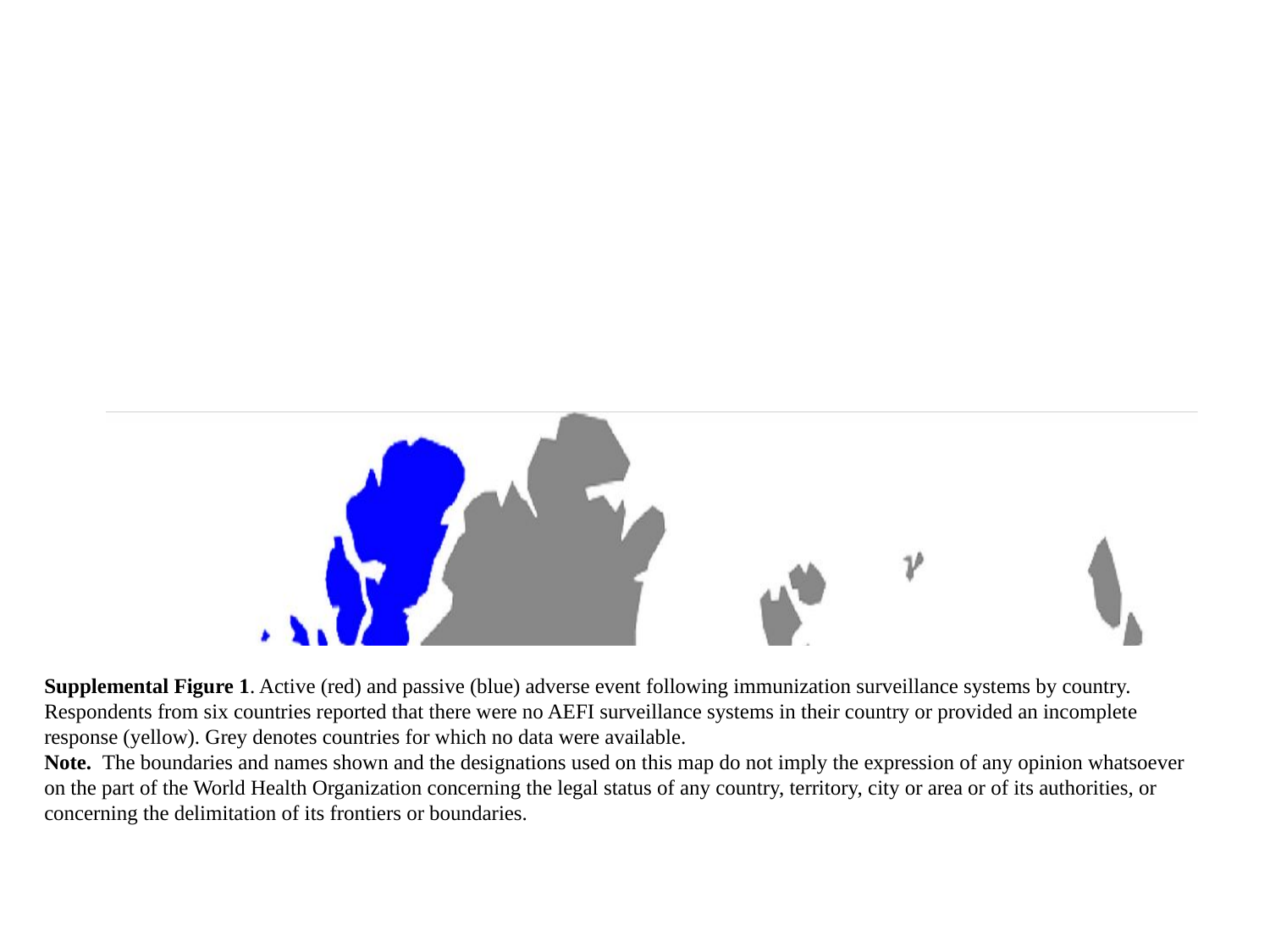

Supplemental Figure 1. Active (red) and passive (blue) adverse event following immunization surveillance systems by country. Respondents from six countries reported that there were no AEFI surveillance systems in their country or provided an incomplete response (yellow). Grey denotes countries for which no data were available.
Note. The boundaries and names shown and the designations used on this map do not imply the expression of any opinion whatsoever on the part of the World Health Organization concerning the legal status of any country, territory, city or area or of its authorities, or concerning the delimitation of its frontiers or boundaries.
